# Dynamics of microcyst-like epithelial changes associated with Belantamab mafodotin therapy in a patient with multiple myeloma—a case report

**DOI:** 10.1038/s41408-024-01110-x

**Published:** 2024-08-07

**Authors:** Lukas Schloesser, Karin U. Loeffler, Annkristin Heine, Frank G. Holz, Martina C. Herwig-Carl

**Affiliations:** 1 Department of Ophthalmology, Medical Faculty, University Hospital Bonn, Bonn, Germany; 2 Division of Ophthalmic Pathology, Department of Ophthalmology, University of Bonn, Bonn, Germany; 3 Department of Oncology, Hematology, Rheumatology and Immunoncology, University Hospital Bonn, Bonn, Germany; 4 Center for Integrated Oncology (CIO), University Hospital Bonn, Bonn, Germany

**Keywords:** Signs and symptoms, Diseases

Dear Editor,

Belantamab mafodotin (Blenrep®; GSK2857916;Glaxo-Smith-Kline, London-Brentford, UK) belongs to a relatively new class of therapeutics characterized by a chemical fusion of monoclonal antibodies to a conventional chemotherapeutic drug which is called antibody-drug conjugates (ADCs) [1]. The rationale is to limit systemic toxicity and to specifically target tumor cells by interaction of monoclonal antibodies with their epitope thereby increasing therapeutic effectivity [2]. Belantamab mafodotin consists of an afucosylated humanized anti-BCMA (B-cell maturation antigen) mouse antibody (mAb) conjugated to the microtubule inhibitor monomethyl auristatin F (MMAF) and binds to FcγRIIIa on plasma cells resulting in antibody-dependent cell-mediated cytotoxicity (ADCC) [3]. Belantamab mafodotin has been approved for patients with advanced multiple myeloma which were already refractory to some of the other drug classes used in the therapy of multiple myeloma [3].

Oncologists and patients may be concerned about the transient corneal changes that may occur during Belantamab mafodotin therapy resulting in microcyst-like epithelial changes (MECs) which can be also caused by other ADCs including depatuxizumab mafodotin (ABT-414) and vorsetuzumab mafodotin (SGN-75) [4–6]. However, ophthalmologists have learned to deal with these corneal changes which are typically fully reversible. Transient deterioration of VA and the corneal status have to be monitored in order to guide the patients through the undulating visual changes during the treatment period and to help oncologists to adjust therapy if needed. Around 70% of the patients treated with Belantamab develop MECs [1]. Over 50% of this group are symptomatic reporting blurred vision and/or symptoms of dry eye syndrome [1]. MECs typically develop in the early phase of treatment after the second cycle of therapy after a median interval of 37 days [1]. Farooq et al. generated a grading system for MECs based on the function (BCVA) and the clinical presentation with respective recommendations regarding therapeutical adjustment [1]. According to this grading system, a grade 2 MECs is reached in case of moderate superficial keratopathy and a vision loss of two or more lines [1]. The authors recommend the interruption of therapy with belantamab until the improvement of vision of at least one line [1]. Therefore, close collaboration between the oncologist and the ophthalmologist as well as frequent monitoring of the course of the disease is required [1].

The current hypothesis is that this side effect is caused by uptake of MMAF by basal corneal epithelial cells by macropinocytosis resulting in formation of microcystoid lesions due to the induction of apoptosis [1]. It is not clear yet whether the ADCs reach the differentiating basal epithelial cells via the limbal blood supply or by the tear film, but the initial pattern of distribution and the consecutive centrifugal movement suggest delivery by the limbal bloodstream [1]. The mechanism of induction of apoptosis in the basal corneal epithelial cells is still not known. On-target toxicity by receptor-mediated endocytosis of the ADC as well as off-target toxicity, which could be mediated by Fc-receptor-mediated endocytosis, macropinocytosis, bystander toxicity, or passive diffusion, could be possible mechanisms of induction of apoptosis [2, 7].

## Case presentation

A 71-year-old female patient was referred to our clinic by the Department of Hemato-Oncology in May 2021 for ophthalmological evaluation before therapy with Belantamab mafodotin for advanced multiple myeloma was initiated. The patient reported a history of dry eye syndrome and moderate glaucoma (without affection of central vision) and presented accordingly with mild blepharitis and mild signs of keratoconjunctivitis sicca, i.e. dry eye syndrome, as well as a beginning corticonuclear cataract in both eyes. In addition, the right eye showed a nevus at the nasal upper eyelid, and the left eye had a paracentral inferior small corneal scar at 5 o’clock. The best-corrected VA was 0.5 (decimal) in both eyes due to lens opafication and dry eye syndrome. The patient was already under topical therapy with dorzolamide drops (preservative-free) 2x per day for glaucoma, preservative-free artificial tears five times daily, and a moisturizing ointment before hours of sleep. The patient provided consent for the use of her anonymized medical data for scientific purposes.

**Fig. 1:**
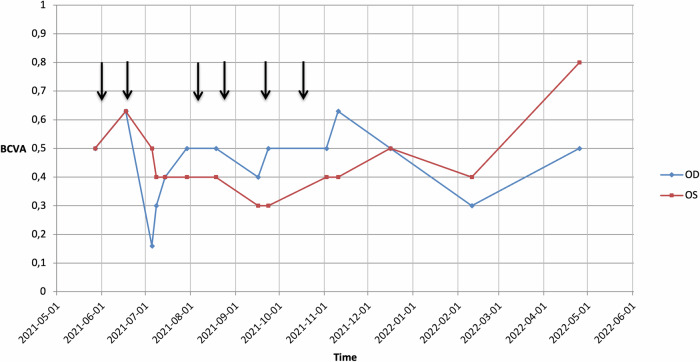
Visual acuity changes related to systemic therapy. Time course of visual acuity during Belantamab mafodotin therapy: rhombuses (right eye, OD, blue line) and triangles (left eye, OS, red line) marking the date of measurement of the best corrected visual acuity (BCVA, decimal), arrows highlighting Belantamab mafodotin administration.

**Fig. 2:**
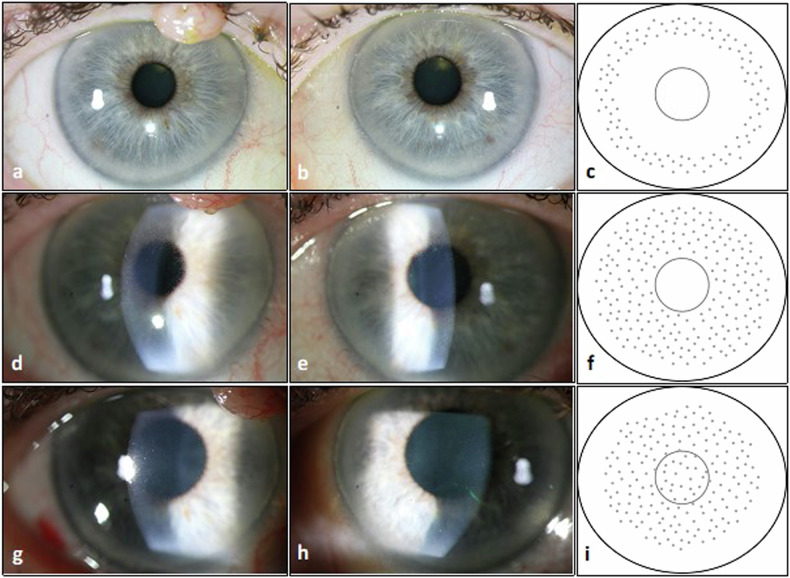
Clinical images. Photographs of the anterior segment and corresponding simplified scheme of distribution of corneal microcystoid lesions (note the eyelid margin nevus at the upper right eyelid visible in photographs **a**, **d**, **g**). The outer black circle represents the limbus and the inner gray circle represents the pupil margin. Each row refers to one consultation. **a**, **b** OD and OS on 2021-06-17, **c** corresponding schematic picture, **d,** **e** OD and OS on 2021-07-05, **f** corresponding schematic picture, **g**, **h** OD and OS on 2021-07-29, **i** corresponding schematic picture.

Our patient received the first administration of Belantamab mafodotin in June 2021 (Fig. 1). Three weeks later, she presented with MECs in the typical circular peripheral pattern with an unaffected central cornea without symptoms or vision loss (Fig. 2a–c). On the following visit (8th of July, before the third planned Belantamab mafodotin administration (13th of July)), we saw a centrifugal movement of lesions towards the corneal center leaving only a small unaffected central area and a respective vision decline of two lines in the right eye (Fig. 2d–f). The patient reported blurred vision with foreign body sensation. We recommended interruption of Belantamab mafodotin administration until partial functional recovery (Fig. 1) as well as to intensify the topical therapy with additional preservative-free moisturizing eye drops five times a day resulting in application of moisturizing eye drops 10× per day. Belantamap mafodotin administration was discontinued and at the next visit (14th of July), the patient showed a partial functional recovery with a vision of 0.4 (decimal) in both eyes, although the MECs affected the complete corneal center with gradual clearing of the periphery (Fig. 2g–i). The visiual acuity remained stable over the following controls and the patient reported moderate symptoms with blurred vision and elevated glare sensitivity. The next four administrations of Belantamab mafodotin (standard dosage) were performed at an interval of three to four weeks between the 3rd of August and the 13th of October 2022 (Fig. 1). The vision remained stable with only minor fluctuations in vision and without worsening of the symptoms.

The cornea status was stable as well with a clear periphery, microcystoid lesions beginning in the mid periphery, and involvement of the corneal center. In the following, the oncologists decided to switch the therapy for the multiple myeloma to the anti-CD38 inhibitor isatuximab due to an inadequate treatment response. We prolonged the intervals between the visits to 1 up to 2 months (2^nd^ of November, 10^th^ of December, 10^th^ of February 2022, and 25^th^ of April 2022). Over all visits, vision was stable fluctuating around baseline vision. Clearing of the peripheral cornea progressed with continuous disappearance of the MECS in the center and finally without any visible cystoid changes at the visit on the 25^th^ of April. The patient reported a relatively sudden disappearance of the reported symptoms three weeks before.

## Discussion

In this patient with multiple myeloma, we were able to observe the pattern of distribution and movement of the MECs starting in the periphery with an initially unaffected corneal center followed by involvement of the entire cornea under Belantamab mafodotin therapy. After discontinuation of Belantamab mafodotin, the MECs slowly disappeared finally leaving only the central cornea affected, which then completely resolved almost 6 months after the last Belantamab mafodotin administration.

The chronological dynamics of the microcystoid lesions following ADC therapy are poorly characterized so far. The DREAMM-2 study reports a median interval of 86.5 days until complete resolution of the microcystoid lesions [1]. In our case, it took almost 6 months until we no longer detected any corneal microcystoid lesions. The sudden disappearance of the subjective symptoms three weeks before the visit seems to be related to the final disappearance of the corneal microcystoid lesions. Our patient suffered from dry eye syndrome prior to Belantamab mafodotin therapy and was under local therapy with dorzolamide two times a day due to primary open-angle glaucoma. This predisposition and the potentially irritating topical antiglaucomatoustherapy probably have been important factors that may have significantly delayed the resolution of the corneal microcystoid lesions. This illustrates the high variability regarding the duration of the presence of MECs depending on the individual conditions and local factors that either support or suppress epithelial regeneration.

The observed pattern of distribution and variation of corneal microcystoid lesions fits the pathophysiologic background with limbal delivery of ADCs through the blood vessels, and induction of apoptosis in basal corneal epithelial cells resulting in the formation of microcystoid lesions and their consecutive movement towards the corneal center. As the epithelial cells derive from limbal stem cells, the renewal of these cells occurs centripetally as well as the disappearance of the MECs. Regarding the documentation of the microcystoid lesions by photography, the best way to visualize the changes is in retroillumination and pharmacological widened pupil that was denied by the patient. Although we prepared precise drawings documenting the distribution of the microcytoid lesions, this has to be taken into account as a limitation of our case report.

Although Belantamab mafodotin lost significance in the armamentarium for the treatment of multiple myeloma, there are other ADCs, especially those containing mafodotin as a cytotoxic component, used in the therapy for other diseases [4, 5]. Corneal side effects have been also reported for ADCs using a cytotoxic component other than mafodotin [5]. In the future, ophthalmological monitoring of patients receiving ADC treatment will be mandatory to guide the patients through episodes of visual deterioration, to identify other related or non-related potentially sight-threatening conditions, to initiate adequate topical treatment, and finally to support the oncologists in their treatment decisions. Nevertheless, clinical management of belantamab mafodotin therapy based on the visual acuity and the pattern of microcystoid changes as described by Farooq et al. can be misleading as visual acuity is influenced by many different factors which can also change throughout therapy (e.g. lens status, retinal findings) [1]. In addition, not all patiens with MECs suffer from a deteriotion of visual acuity. On the one hand, patients with other reasons for a decline in visual acuity or persistent deterioration of vision after resolution of the microcyst-like epithelial changes should get the potentially life-saving therapy with belantamab mafodotin (or at least in a reduced dosage) despite a decline in BCVA of 2 or more lines (recommendation of the DREAMM-2 by Farooq et al.) [1]. On the other hand, patients who lose abilities of daily living (e.g. reading) in a palliative setting should possibly no longer receive therapy with belantamab mafodotin regardless of the objectifiable loss of vision.
